# Macrofollicular Variant of Follicular Thyroid Carcinoma (MV-FTC) with a Somatic *DICER1* Gene Mutation: Case Report and Review of the Literature

**DOI:** 10.1007/s12105-020-01208-1

**Published:** 2020-07-25

**Authors:** L. Samuel Hellgren, Martin Hysek, Kenbugul Jatta, Jan Zedenius, C. Christofer Juhlin

**Affiliations:** 1 Department of Oncology-Pathology, Karolinska Institutet, Stockholm, Sweden; 2 Department of Pathology and Cytology, Karolinska University Hospital, Stockholm, Sweden; 3 Department of Molecular Medicine and Surgery, Karolinska Institutet, Stockholm, Sweden; 4 Department of Breast, Endocrine Tumors and Sarcoma, Karolinska University Hospital, Stockholm, Sweden

**Keywords:** Follicular thyroid carcinoma, Macrofollicular variant, MET, DICER1, Mutation

## Abstract

Benign thyroid lesions such as multinodular goiter and adenomatoid nodules are well-circumscribed lesions displaying a macrofollicular growth pattern and lack of nuclear atypia. The highly unusual macrofollicular variant of follicular thyroid carcinoma (MV-FTC) mirrors these attributes and is thereby misclassified by cytological examination of fine-needle aspiration biopsies. The MV-FTC diagnosis is instead suggested following histological investigation, in which malignant attributes, most commonly capsular invasion, are noted. The bulk of MV-FTCs described in the literature arise in younger female patients and carry an excellent prognosis. A recent coupling to mutations in the *DICER1* tumor suppressor gene has been proposed, possibly indicating aberrancies in micro-RNA (miRNA) patterns as responsible of the tumorigenic process. We describe the cytological, histological and molecular phenotype of a 35 mm large MV-FTC arising in the right thyroid lobe of a 33-year-old female with a family history of multinodular goiter. The tumor was encapsulated and strikingly inconspicuous in terms of cellularity and atypia, but nevertheless displayed multiple foci with capsular invasion. A next-generation molecular screening of tumor DNA revealed missense variants in *DICER1* (p. D1709N) and *MET* (p. T1010I), but no established fusion gene events. After sequencing of germline DNA, the *DICER1* mutation was confirmed as somatic, while the *MET* variant was constitutional. The patient is alive and well, currently awaiting radioiodine treatment. This MV-FTC mirrors previous publications, suggesting that these tumors carry a favorable prognosis and predominantly arise in younger females. Moreover, *DICER1* mutations should be considered a common driver event in the development of MV-FTCs.

## Introduction

Fine-needle aspiration biopsy (FNAB) followed by cytological examination is the gold standard technique for evaluating thyroid nodules, and the analysis provides an estimate of the risk of malignancy and therefore strongly influences the clinical handling of the individual patient. The reporting is recommended to follow a standardized, category-based system entitled “The Bethesda System for Reporting Thyroid Cytopathology”, in which the cytology report is assigned to one of six different categories ranging from 0 to VI, where 0 is “non-diagnostic or unsatisfactory” and VI is “malignant” [1]. As the majority of thyroid lesions in the clinical setting are solitary colloid nodules and cases of multinodular goiter, it is only logical that the Bethesda II class (“benign”) is the most commonly reported category among US institutions, reaching > 70% of all FNABs performed [2]. The risk of malignancy in this category ranges from 0 to 3%, meaning that virtually all cases diagnosed as Bethesda II are benign. Thus, surgery is only advocated if the patient would benefit from a relief of compression of nearby structures. The cytological attributes of a Bethesda II lesion include the detection of abundant colloid deposits and loose formations of follicular-patterned thyrocytes without cellular atypia and nuclear changes associated with papillary thyroid carcinoma (PTC).

The macrofollicular variant of follicular thyroid carcinoma (MV-FTC) is an exceedingly rare thyroid tumor, of which only four cases have been published prior to this case report [3–5]. As the name implies, this lesion is built-up by an encapsulated, macro-follicular mass with tumor cells lacking PTC-related nuclear changes. So far, all MV-FTCs described to this date displayed macrofollicles constituting > 70% the tumor mass, and all cases exhibited limited capsular invasion, but not angioinvasion [3]. Given the rarity of the diagnosis, no definite criteria for MV-FTC seem to exist, but the definition of a macrofollicular variant of PTC is based upon the histological findings of > 50% of follicles arranged as macrofollicles (measuring > 200 µm in diameter) [1]. The hypo-cellularity of the MV-FTC is strongly reminiscent of a colloid nodule, a feature that is reflected by the colloid-like appearance upon gross examination. From a FNAB perspective, these tumors mirror many aspects of a benign thyroid nodule, and the majority of samples are therefore categorized as Bethesda II. From a molecular standpoint, two out of four MV-FTCs were found to carry double somatic mutations of the *DICER1* gene, although the authors were not able to elucidate whether these mutations occurred in *cis* or *trans* [3]. If indeed bi-allelic, the mutations strongly support an abolished tumor-suppressor function of *DICER1* as an important genetic mechanism underlying the development of MV-FTCs. The *DICER1* gene is located on chromosome 14q32 and constitutes a key regulator of micro-RNA (miRNA) maturation from pre-existing pre-miRNAs [6]. Germline mutations in *DICER1* cause the DICER1 syndrome, a multi-tumor condition in which the afflicted family member exhibits an increased risk of pleuropulmonary blastoma, cystic nephroma, ovarian sex cord-stromal tumors (most commonly Sertoli-Leydig cell tumors), various sarcomas as well as multinodular goiter [7–9]. Somatic *DICER1* mutations are also found in small subsets of follicular thyroid adenomas (FTAs), FTCs, PTCs and poorly differentiated thyroid carcinomas (PDTCs), most notably in pediatric or adolescent patients [10–14]. The mutations are believed to disrupt the maturation process of miRNAs and hence drive the oncogenic transformation [6, 11]. Even in the absence of inactivating mutations, *DICER1* gene expression is reduced in the majority of follicular thyroid tumors, a process that has been linked to aberrant expression of the *GABPA* transcription factor. The reduction in *DICER1* levels in turn results in an impaired processing of specific thyroid-related miRNAs and stimulated proliferation [14].

In this report, we describe an MV-FTC with a somatic *DICER1* mutation occurring in a young female patient and detail the cytological and histological work-up of this rare tumor example.

## Case Report

### Clinical Background

**Fig. 1:**
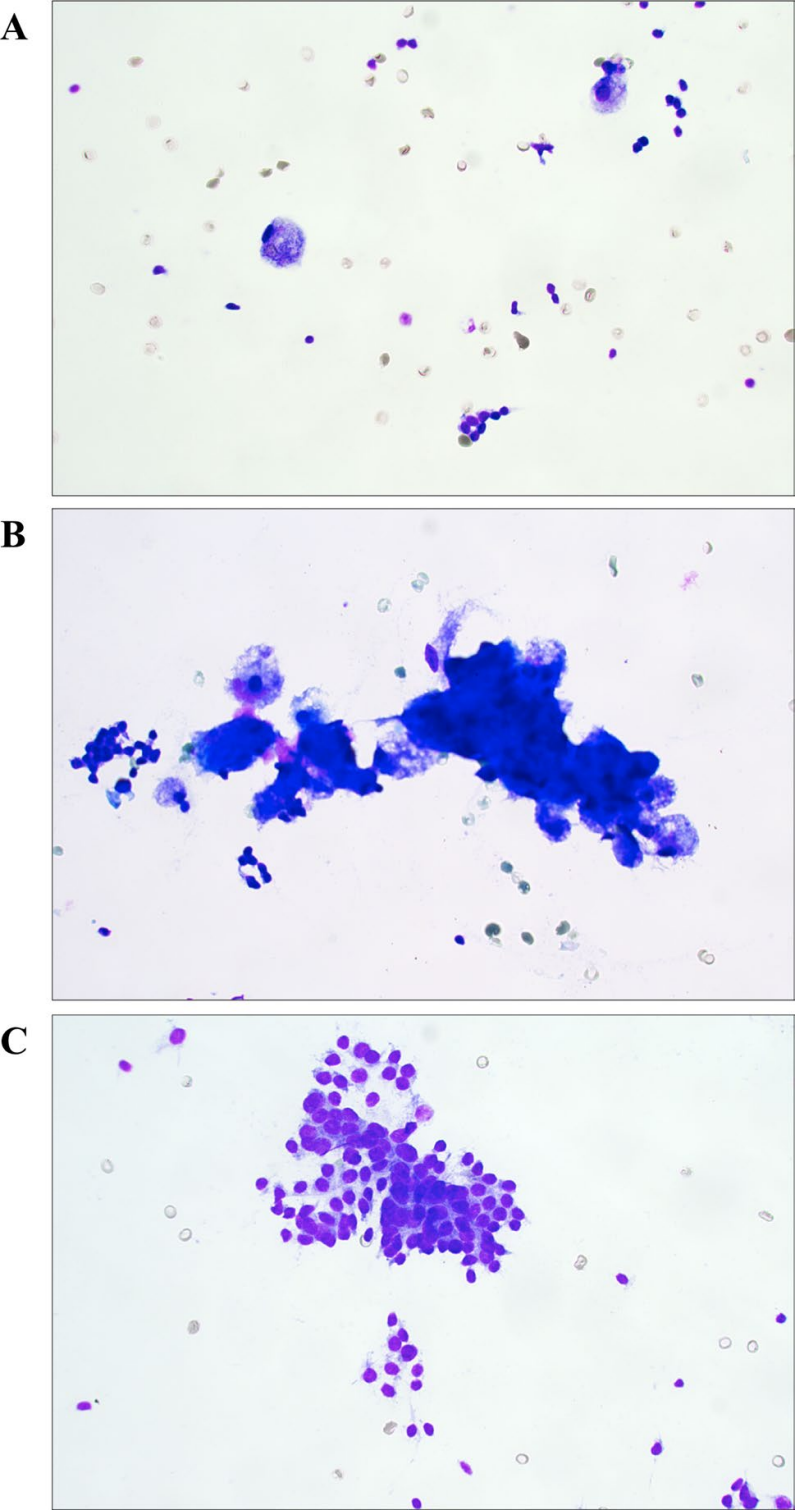
Cytological features of the macrofollicular variant of follicular thyroid carcinoma (MV-FTC). **a** Photomicrograph depicting the main cytological findings of the preoperative fine-needle aspiration biopsy (FNAB); erythrocytes, occasional macrophages and scattered epithelial cells without atypia. **b** Thick colloid matter and infrequent groups of epithelial cells lacking atypia, consistent with a Bethesda II category. **c** Scarce groups of microfollicular-patterned thyroid epithelial cells, vaguely suspicious for a tumorous lesion. These observations were not reported in the original cytology report, but evident upon re-examination. All photomicrographs represent May-Grünwald-Giemsa (MGG) stained slides at ✕ 400 magnification

The patient was a 33-year-old female without known medical conditions, exhibiting a family history of multinodular goiter. In 2016, she saw her general practitioner (GP) for a mild upper respiratory tract infection, with symptoms including a sore throat and cough. The GP identified a right-sided 20 mm firm thyroid nodule upon clinical examination, and ultrasonographic investigations detailed a 25 mm large, partly cystic lesion in the caudal aspect of the right thyroid lobe. An FNAB was undertaken, and the cytology described abundant colloid, macrophages and scarce follicular epithelial structures without atypia. The lesion was therefore considered benign (Bethesda II). The patient was clinically euthyroid, and thyroid tests were normal. Two years later, following an uneventful pregnancy, the patient again saw her GP as the thyroid lesion had increased in size. At this point, the patient was referred to our endocrine surgery department. An ultrasonogram appreciated the size of the nodule to 35 mm. A second FNAB was performed, and the material consisted of thin colloid and rare follicular-patterned thyrocytes, again suggestive of Bethesda II (Fig. 1a–c). As the patient was without symptoms, she was discharged. During the winter of 2019, the patient experienced intensified cervical discomfort that was aggravated in the supine position. She again consulted her surgeon, and a right-sided hemithyroidectomy was performed in early 2020.

### Histopathology

**Fig. 2:**
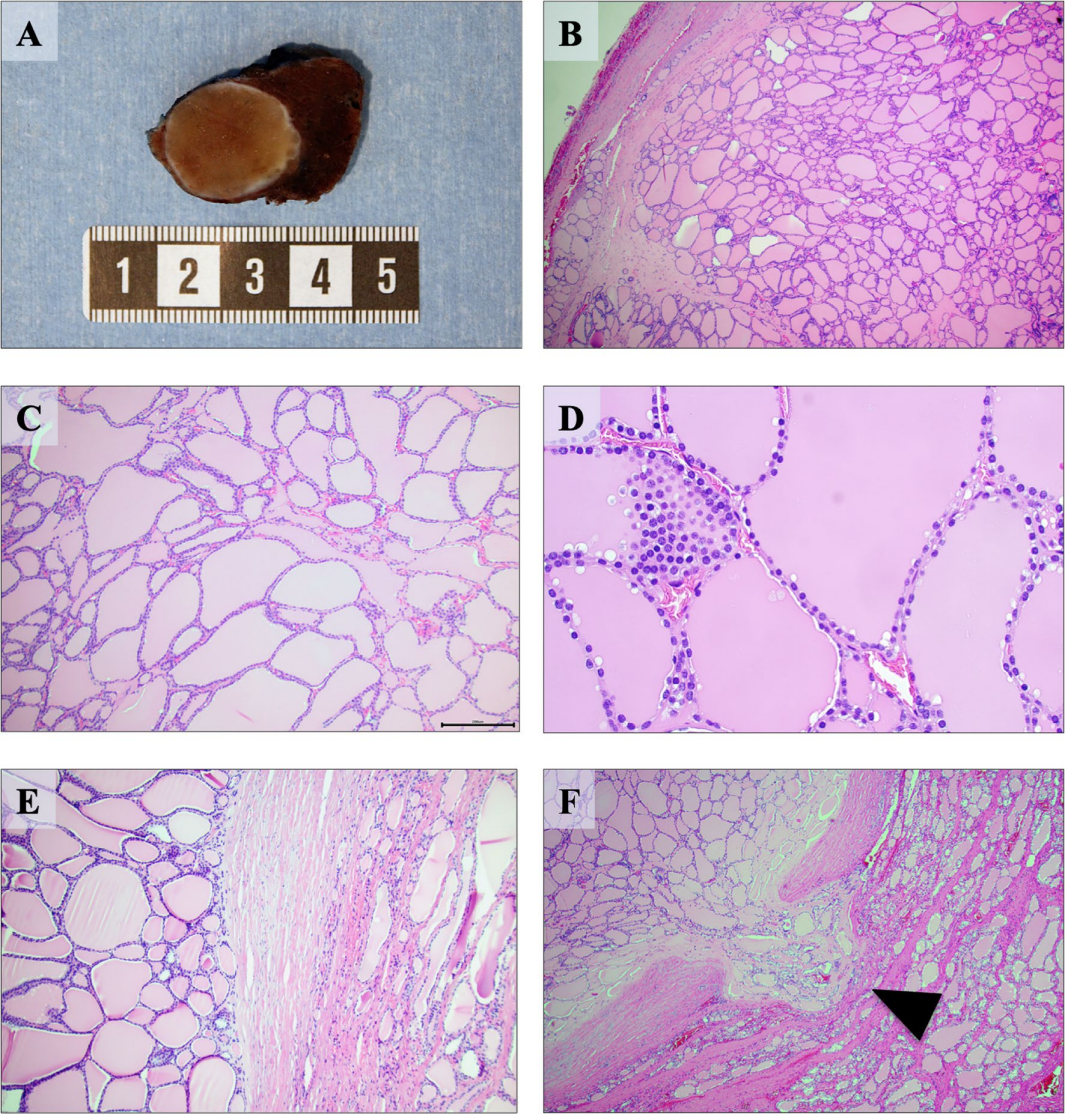
Macroscopic and histological attributes of the macrofollicular variant of follicular thyroid carcinoma (MV-FTC). **a** Cross-section of the gross specimen revealing a well-circumscribed, light brown lesion with a homogenous appearance. Note the macroscopically visible areas with capsular engagement. **b** Hematoxylin and eosin (H&E) stain at low power (✕ 40) magnification visualizing the circumscribed tumor with colloid-like, macrofollicular appearance. **c** H&E stain displaying the macrofollicular growth pattern at ✕ 100 magnification. There was a general lack of degenerative changes, which are usually seen in colloid nodules. Macrofollicles were defined as > 200 µm, which equals the size of the micron bar in the lower right corner. **d** H&E stain at high power (✕ 400) magnification displaying cellular details of the MV-FTC. Note the monotonous-looking nuclei lacking atypia lining the macro-follicles, with occasional, more hypercellular areas. **e** H&E stain at ✕ 100 magnification highlighting the broad, fibrous tumor capsule. Normal thyroid tissue is seen to the right of the image. **f** Clear-cut capsular invasion with a broad, mushroom-like protrusion into the surrounding thyroid parenchyma (arrowhead). Several areas with capsular invasion were noted, but vascular invasion was absent

The lobe weighted 18.3 g and measured 55 × 35 × 30 mm. After sectioning, a 35 mm large nodule was visualized on gross inspection, strongly reminiscent of a colloid nodule (Fig. 2a). Intriguingly, the lesion was seen with a thin fibrous capsule, and occasional areas suggestive of gross infiltration were noted. Histopathological examination revealed a well-circumscribed macrofollicular-patterned lesion suggestive of a colloid nodule, as the specimen was made up of 70–80% macrofollicles with abundant colloid (defined as measuring > 200 µm in diameter) intermingled with scattered follicles of normal sizes (Fig. 2b–e). Degenerative changes usually associated to colloid nodules, such as hemorrhage, fibrosis and calcifications, were not evident. In two areas, the tumor cells protruded through the entire width of the capsule (Fig. 2f), evident of malignant behavior. Pseudo-invasion due to previous FNAB procedures was ruled out due to the absence of associated inflammation and fibrosis. Tumor cells were flat to partially cuboidal, with normochromatic nuclei lacking atypia and PTC-associated changes. Positive immunohistochemical stains included TTF1, PAX8, thyroglobulin and CK19. Negativity was noted for HBME1, V600E mutation-specific BRAF and Pan-TRK (total levels of tyrosine receptor kinase proteins A, B and C). The Ki-67 index was 5.5%. All immunohistochemical analyses were carried out in our accredited pathology laboratory at Karolinska University Hospital, Stockholm, Sweden using a Ventana Benchmark Ultra system (Ventana Medical Systems, Tucson, AZ, USA). The diagnosis was consistent with an MV-FTC. No extrathyroidal extension was noted, and the TNM cancer staging was denoted as pT2Nx. Surgical margins were negative. A completion lobectomy was performed, and the excised specimen lacked pathological findings. The patient is currently well and planned for radioiodine ablation therapy with 1.1 gigabecquerel (GBq).

### Cytological, Histological and Molecular Work-up of the Tumor Tissue

Given the rarity of MV-FTCs, re-investigations of the previous biopsies and histology were performed, and several comprehensive molecular investigations were launched. Cytological and histological preparations were reviewed via light microscopy, and the re-evaluation was performed by three of the authors: MH reexamined the cytology while LSH and CCJ reviewed the histology. DNA from formalin-fixated paraffin-embedded tumor tissue was extracted and interrogated using the Oncomine Solid Tumor Panel (Ion Torrent S5, Hi-Q Chef; Thermo Scientific, Waltham, MA, USA), the Oncomine Childhood Cancer Research Assay (Thermo Fisher Scientific, Waltham, MA, USA) as well as bi-directional Sanger sequencing of the *TERT* promoter region. The Oncomine Solid Tumor Panel screens for > 1800 mutations in 22 cancer-associated genes, but no mutations in any of these genes were observed. Moreover, given the previous knowledge that promoter mutations in *TERT* are associated with worse prognosis in follicular thyroid tumors, bi-directional Sanger sequencing of the promoter region was also launched [15–17]. The methodology has been previously described [18]. No *TERT* promoter mutation was observed.

Following these initial absent findings, we expanded our investigations using the Oncomine Childhood Cancer Research Assay, a panel that screens for mutations in 126 genes (full exon coverage of 44 genes and hotspot mutational screening of additional 82 genes), copy number alterations of 24 genes and > 1700 gene fusion variants in 88 genes. Both the Oncomine Solid Tumor Panel and the Oncomine Childhood Cancer Research Assay are used in clinical routine at our department, and the bioinformatics and associated interpretations were performed by one of the authors (KJ). By the extended investigation, we detected an exon 26 c.5125 G > A (p. D1709N) missense variant in the *dicer 1 ribonuclease III* (*DICER1*) gene and an exon 14 c.3029 C > T (p. T1010I) missense variant in the *MET proto-oncogene receptor tyrosine kinase* (*MET*). The genotypic information for both variants is detailed in Table 1. Both variants are enlisted in the Catalogue of Somatic Mutations in Cancer (COSMIC) database. The *DICER1* variant D1709N locates to the functionally important RNAse IIIb domain of the mature DICER1 protein, and this specific variant is reported as a somatic mutation in several DICER1 syndrome-associated tumor forms, including ovarian Sertoli-Leydig cell tumors, pleuropulmonary blastoma and single cases of pediatric thyroid cancer [19–22]. Interestingly, the D1709N variant has been functionally proven to cause a significant reduction in 5-p-miRNAs, leading to a tumor-propagating phenotype in cell lines [23]. The *MET* variant p. T1010I is interpreted as pathogenic according to the COSMIC database, and considered oncogenic when mutated on the somatic level in small cell lung cancer, as the authors observed enhanced tumorigenicity when investigating this variant from a functional perspective [24].

**Table 1:** Detailed information of the MET and DICER1 variants detected in tumor DNA via next-generation sequencing

Gene name	Chr.	Variant type	Coding DNA variant	Amino acid change	FATHMM prediction	Present in germline	Global MAF*	Germline prediction#
*MET*	7q31.2	Missense	c.3029 C > T	p. T1010I	Pathogenic	Yes	0.00339	Conflicting
*DICER1*	14q32.13	Missense	c.5125 G > A	p. D1709N	Pathogenic	No	n.d.	n.a.

*Chr* Chromosome,* FATHMM* Functional Analysis through Hidden Markov Models,* n.d.* not determined,* n.a.* not applicable

*Global minor allele frequency of this variant as according to ClinVar, the NCBI online resource

#In silico prediction of the variant as according to ClinVar, “conflicting” denote uncertain pathogenicity regarding a constitutional variant

### Validation of Mutations in Constitutional Tissues

To verify the findings from the tumor DNA screening, the patient consented to germline mutational testing using leukocyte DNA extracted from peripheral blood. The sequencing was performed using a standardized next-generation platform at the Department of Clinical Genetics at the Karolinska University Hospital. In short, the *DICER1* variant was not detected in leukocyte DNA and thereby proved as somatic, while the T1010I *MET* variant was identified and hence classified as constitutional (Table 1).

## Discussion

Detailed morphological and genetic reports of rare variants of thyroid cancer could potentially provide important clues regarding prognostication and underlying etiology. This is not least exemplified by the exceedingly uncommon MV-FTC entity. To our knowledge, even though the lesion described herein merely constitutes the fifth case of such a tumor presented in the scientific literature, several common denominators with previous cases were identified. Most strikingly, it seems as though MV-FTCs predominantly arise in adolescent to middle-aged euthyroid female patients [3]. Preoperatively, the lesions are commonly misjudged as benign, and surgery was often performed following extended nodular growth. The MV-FTCs reported so far had a diameter of > 2 cm but ≤ 4 cm, and histological examinations of these lesions report a predominant macrofollicular growth pattern and signs of capsular, but not vascular, invasion. Importantly, from a prognostic standpoint, all MV-FTC cases lack evidence of relapsing disease [3]. However, in our own experience, subsets of FTCs may display a focal macrofollicular component, and single cases have been shown to metastasize [18]. Moreover, rare reports of macrofollicular variants of PTCs, a lesion most often associated to an idle clinical course, have been associated to the development of distant metastases [25]. Although these observations somewhat contradict the assumption that most malignant thyroid tumors with macrofollicular growth patterns are clinically indolent, it seems safe to conclude that the bulk of cases generally exhibit a benign clinical course. As of this, it is imperative for the practicing pathologist to adhere to strict criteria when considering an MV-FTC diagnosis, and given the previous publications, a cut-off of > 70% macrofollicles might be appropriate for diagnosing this unusual and seemingly indolent tumor type [3].

From a cytological perspective, MV-FTCs seem to be preoperatively diagnosed as benign through FNAB [3], which is not entirely surprising given the macrofollicular appearance. As a FNAB diagnosis of a Bethesda IV category lesion (“follicular neoplasm” or ”suspicious for a follicular neoplasm”) demands a predominant cell population arranged in microfollicular or trabecular formations with scant or absent colloid, this definition will most likely render MV-FTCs falsely annotated as Bethesda II lesions [1]. If misdiagnosed as colloid nodules, subsets of MV-FTCs could in theory recur as distant metastases in the future if left untreated—even though the prognosis seems to be excellent for the five cases identified up-front. Even so, all assumptions would be based on five cases correctly identified as MV-FTCs postoperatively. Hence, our collective knowledge of this entity is very limited, and no deviations from the current guidelines can be recommended at this stage. Retrospective studies of patients with metastatic deposits and previous benign cytology reports or a postoperative diagnosis of multinodular goiter could probably help us elucidate the true frequency of the MV-FTCs. Indeed, previous reports indicate that small subsets of seemingly benign thyroid nodules might give rise to metastatic disease, hypothetically highlighting the clinical importance to recognize the MV-FTC entity [26].

Somatic *DICER1* mutations have been previously reported in well-differentiated thyroid carcinomas such as FTCs and PTCs, and the specific p. D1709N *DICER1* mutation found in our MV-FTC case has been previously reported in a single case of PTC [22]. This variant is located in the functionally important RNAse IIIb domain and constitutes a somatic hotspot mutation in DICER1 syndrome-associated tumors [6]. Missense *DICER1* mutations of the RNAse IIIb region are thought to be oncogenic, and tumors with this alteration exhibit reduced levels of 5p miRNAs, whereas truncating mutations have more global effects on miRNA processing [27, 28]. Interestingly, DICER1 syndrome patients often carry deleterious germline *DICER1* mutations, and develop somatic, second-hit type missense mutations in hotspot regions of the RNAse II domain. Although this bi-allelic model is in line with the Knudson two-hit hypothesis indicating a *bona fide* tumor suppressor function, there are instances in for example cases of Wilms tumors in which *DICER1* RNAse III mutations are seen without associated second hits (in terms of a *trans* mutation or loss of the remaining allele) [28]. Moreover, data is accumulating that *DICER1* can function as a haploinsufficient tumor suppressor, in which a single mutational event would suffice for tumor development [29]. As of this, we cannot exclude a tumor-propagating role of the single p. D1709N *DICER1* mutation found in our study.

Interestingly, out of the other four published MV-FTC cases, two of these carried double *DICER1* mutations: an exon 16 splice site mutation plus a codon 1705 hotspot mutation in one case, as well as an exon 24 frameshift alteration and a codon 1810 hotspot mutation in the second case [28]. The two other MV-FTCs exhibited *DICER1* wildtype sequences. Hence, out of the five reported MV-FTC cases, a total of three carried somatic *DICER1* mutations located in exons 25 or 26 of the functionally important RNAse IIIb region. In all, this strongly supports the theory that these tumors develop because of dysregulated *DICER1* function and miRNA maturation, and future studies might help elucidate if dysregulation of miRNA through molecular aberrancies of *DICER1* affects the characteristic colloid-rich, macrofollicular growth patterns observed within this tumor type. Interestingly, previous studies show that *DICER1* is required for thyroid follicular organization and thyrocyte differentiation during the embryonic stage, and the establishment of thyroid follicles is partly regulated by a concerted action of several miRNAs [30, 31]. Moreover, the established association between multinodular goiter and the DICER1 syndrome further substantiates the association between *DICER1* mutations and the cystic “ballooning” of colloid. To our knowledge, *DICER1* mutated thyroid tumors have not yet been systematically reviewed from a histological standpoint in terms of the overall follicular volume, but would constitute a potentially interesting follow-up study given the accumulated evidence suggesting a linkage between follicular development and *DICER1* function.

The constitutional *MET* variant has been previously shown to positively influence invasive properties of breast cancer cell lines [32], and has previously been described on both the somatic and constitutional level in thyroid carcinomas, including FTCs [33]. Nevertheless, the allele frequency and overall pathogenicity of this variant in the germline setting is still debatable according to the ClinVar, a public archive of human genetic variations and associated phenotypes. Therefore, we are not in a position to denote this variant as a pathogenic mutation, but rather as a fairly unusual single nucleotide variant of unknown significance. Whether or not the T1010I *MET* variant in any way influenced the development of this MV-FTC remains to be investigated.

## Conclusions

To summarize, we present the rare occurrence of an MV-FTC and further substantiate the association between this unusual tumor type and *DICER1* hotspot mutations. As no other somatic mutation or gene fusion was observed, this could potentially suggest a driving status of this mutation in the development of this exceedingly rare variant of follicular thyroid carcinoma.

## Data Availability

The data that support the findings of this study are included within the article itself.
